# Specific neuropsychiatric symptoms are associated with functional decline trajectories in Alzheimer’s disease and Lewy body dementia: a five-year follow-up study

**DOI:** 10.3389/fmed.2023.1267060

**Published:** 2023-10-17

**Authors:** Miguel Germán Borda, Kolbjørn Kallesten Brønnick, Elkin Garcia-Cifuentes, Alberto Jaramillo-Jimenez, Carlos Reyes-Ortiz, Jonathan Patricio-Baldera, Hogne Soennesyn, Mario Ulises Pérez-Zepeda, Audun Osland Vik-Mo, Dag Aarsland

**Affiliations:** ^1^Centre for Age-Related Medicine (SESAM), Stavanger University Hospital, Stavanger, Norway; ^2^Semillero de Neurociencias y Envejecimiento, Ageing Institute, Medical School, Pontificia Universidad Javeriana, Bogotá, Colombia; ^3^Division of Clinical Geriatrics, Department of Neurobiology, Care Sciences and Society, Karolinska Institutet, Stockholm, Sweden; ^4^Faculty of Health Sciences, University of Stavanger, Stavanger, Norway; ^5^Departamento de Neurología, Unidad de Neurociencias, Hospital Universitario de San Ignacio, Bogotá, Colombia; ^6^Grupo de Neurociencias de Antioquia, School of Medicine, Universidad de Antioquia, Medellín, Colombia; ^7^Institute of Public Health, College of Pharmacy and Pharmaceutical Sciences, Florida A&M University, Tallahassee, FL, United States; ^8^Escuela de estadística de la Universidad Autónoma de Santo Domingo, Santo Domingo, Dominican Republic; ^9^Instituto Nacional de Geriatría, Dirección de Investigación, Mexico City, Mexico; ^10^Centro de Investigación en Ciencias de la Salud (CICSA), FCS, Universidad Anáhuac México Campus Norte, Huixquilucan, Estado de Mexico, Mexico; ^11^Department of Clinical Medicine, University of Bergen, Bergen, Norway; ^12^Department of Old Age Psychiatry, Institute of Psychiatry, Psychology and Neuroscience, King’s College London, London, United Kingdom

**Keywords:** dementia, functionality, Alzheimer’s disease, behavioral disturbances, Lewy body dementia, neuropsychiatric symptoms

## Abstract

**Background:**

Neuropsychiatric symptoms (NPS) are often overlooked and under-identified symptoms associated with dementia, despite their significant impact on the prognosis of individuals living with the disease. The specific role of certain NPS in functional prognosis remains unclear.

**Aims:**

To determine the association of different NPS with functional decline in people living with Alzheimer’s disease (AD) or Lewy body dementia (LBD).

**Methods:**

This is an analysis of data from the Dementia Study of Western Norway (DemVest) with 196 patients included of which 111 had AD and 85 LBD. The Neuropsychiatric Inventory (NPI) and the Rapid Disability Rating Scale (RDRS-2) for activities of daily living were administered annually for 5 years. NPI total score and individual items with RDRS-2 trajectories were analyzed with linear mixed models.

**Results:**

The LBD group exhibited higher levels of functional impairment and a greater burden of NPS at baseline. Over the 5-year follow-up, hallucinations, aggression, depression, anxiety, apathy, disinhibition, aberrant motor behavior, nighttime behavior disturbances, and abnormal eating patterns were significantly associated with the decline in functional abilities in individuals with AD, as well as irritability and aberrant motor behavior in those with LBD.

**Discussion:**

These results highlight the relevance of early detection and intervention of these particularly relevant NPS, due to its potential of also impacting physical function. Better detection and management of these NPS could improve functional prognosis in people living with dementia.

**Conclusion:**

Specific NPS demonstrate relevant distinct associations with Longitudinal trajectories of functional decline in AD and LBD.

## Introduction

Increasing life expectancy has led to a demographic shift, where the older population is growing faster than other age groups (1). This trend is expected to persist in the upcoming years, with a rise in the prevalence of chronic diseases and the incidence of age-related complex conditions, such as dementia (2). In fact, Alzheimer’s disease (AD) is the most frequent neurodegenerative dementia, followed by Lewy body dementia (LBD) (3). AD and LBD have distinctive features, heterogeneous trajectories, and multiple factors influencing their course (i.e., cognitive, physical, social, and environmental), resulting in varied clinical phenotypes and decline profiles within the same type of dementia (4–6). Functional decline is a key marker of disease progression and a relevant outcome in all dementia cases. However, it is not solely the result of cognitive deficits; but also depends on various non-cognitive factors, such as neuropsychiatric symptoms (NPS) (7). Evidence shows that NPS might influence disability rates, producing deleterious consequences such as poor quality of life, higher morbidity, and dependency (8).

NPS are present in all types of dementia and have been associated with negative outcomes, such as poor treatment response, increased caregiver burden, and a higher risk of developing other diseases and geriatric syndromes (9). Moreover, NPS (e.g., delusions, hallucinations, depression, apathy) play an important role in the functional prognosis of people living with dementia (7, 9).

Our group has recently shown that the total burden of NPS is independently associated with faster functional decline in individuals with AD or LBD. However, our results also exhibited relevant differences between functional impairment in AD and LBD, with a constant and more pronounced decline in the latter (7). Current evidence about the impact of specific NPS on activities of daily living (ADL) decline is scarce, especially in those diagnosed with LBD (10–12). Previous studies on AD have reported an association between apathy, psychotic symptoms, and functional decline, but the reproducibility of these conclusions should be further examined in external samples (13). Therefore, this study aims to determine the association of specific NPS with the trajectories of functional decline in people living with AD and LBD in the mild stage and across a 5-year follow-up.

## Materials and methods

### Setting and participants

This is a longitudinal analysis of a Norwegian cohort study with yearly assessment of patients referred to dementia clinics in Hordaland and Rogaland counties, entitled ‘The Dementia Study of Western Norway’ (DemVest) (5). We included patients with mild dementia, defined as a Mini-Mental State Examination (MMSE) score ≥ 20 or a Clinical Dementia Rating (CDR) global score = 1 (12, 14). The diagnosis of dementia was established according to the DSM-IV criteria and further classified as a specific type of dementia when complying with the corresponding validated criteria (15–17). DLB and PDD were merged into the LBD group based on their clinical and pathological similarities (18). Pathological diagnosis was ascertained in the DemVest study, and its congruency with the clinical diagnostic was above 80% (5). Exclusion criteria were moderate or severe dementia, delirium, previous bipolar or psychotic disorder, terminal illness, or a recently diagnosed major somatic disease, which could significantly impact cognition, function, or study participation. Further information regarding the DemVest study can be found elsewhere (5, 19).

For the current analysis, we included patients with AD (*n* = 111) and LBD (*n* = 85), yielding a total of 196 participants (power 87%). We included data registered annually over a 5-year follow-up (see Table 1).

**Table 1:** Descriptive analysis of the sample by diagnosis.

	AD						LBD					
	Baseline	1	2	3	4	5	Baseline	1	2	3	4	5
Mean (SD)/*n* (%)	111	111	98	91	77	51	85	85	71	55	38	20
Age	75.20 (7.72)						75.42 (6.9)					
Sex (women)	81 (72.97)						38 (44.71)					
CIRS	5.33 (2.44)	5.33 (2.44)	5.22 (2.43)	5.16 (2.44)	4.93 (2.35)	4.90 (2.06)	6.22 (2.52)	6.0.22 (2.52)	6.28 (2.42)	6.43 (2.27)	6.44 (2.22)	6.55 (2.13)
MMSE	23.60 (2.31)	21.38 (3.88)	18.65 (5.13)	15.56 (6.32)	13.11 (7.43)	11.87 (8.17)	23.65 (3.20)	21.58 (5.32)	17.5 (7.37)	15.97 (8.05)	16.22 (7.27)	16.5 (8.08)
Function	1.58 (0.47)	1.98 (0.59)	2.24 (0.58)	2.60 (0.60)	2.86 (0.616)	2.92 (0.532)	1.95 (0.64)	2.32 (0.75)	2.80 (0.69)	3.00 (0.62)	2.97 (0.63)	3.20 (0.52)
Total NPI	3.53 (2.65)	3.55 (2.38)	3.86 (2.67)	4.20 (2.35)	4.36 (2.43)	4.35 (2.28)	4.70 (2.51)	4.28 (2.62)	4.21 (2.32)	4.50 (2.65)	4.55 (2.42)	5.05 (2.64)
Delusions	31 (27.93)	27 (24.32)	31 (31.63)	29 (31.87)	23 (29.87)	17 (33.33)	31 (36.47)	33 (38.82)	24 (33.80)	19 (34.55)	12 (31.58)	8 (40)
Hallucinations	10 (9.01)	10 (9.01)	17 (17.35)	19 (20.88)	18 (23.68)	14 (27.45)	50 (58.82)	44 (51.76)	37 (52.11)	32 (58.18)	20 (54.05)	13 (65)
Agitation/aggression	30 (27.03)	30 (27.03)	27 (27.55)	31 (34.07)	36 (46.75)	25 (49.02)	24 (28.24)	18 (21.18)	22 (30.99)	21 (38.18)	12 (31.58)	10 (50)
Depression/dysphoria	61 (54.95)	66 (59.46)	51 (52.04)	54 (59.34)	41 (53.25)	26 (50.98)	53 (62.35)	46 (54.12)	37 (52.11)	28 (50.91)	25 (65.79)	14 (70)
Anxiety	39 (35.14)	29 (26.13)	35 (36.08)	35 (38.46)	34 (44.16)	22 (43.14)	40 (47.06)	32 (47.06)	29 (40.85)	17 (30.91)	15 (39.47)	9 (45)
Euphoria	7 (6.31)	7 (6.31)	7 (7.14)	9 (10)	7 (9.09)	6 (11.76)	1 (1.18)	2 (2.35)	2 (2.82)	4 (7.27)	4 (10.53)	2 (10)
Apathy	52 (46.85)	61 (54.95)	59 (60.2)	49 (53.85)	46 (59.74)	31 (60.78)	52 (61.18)	59 (69.41)	40 (56.34)	30 (54.55)	17 (44.74)	8 (40)
Disinhibition	22 (19.82)	27 (24.32)	25 (25.51)	25 (27.47)	24 (31.17)	18 (35.29)	17 (20)	22 (25.88)	19 (26.76)	14 (25.45)	9 (23.68)	5 (26.32)
Irritability	45 (40.54)	48 (43.24)	42 (42.86)	41 (45.05)	32 (41.56)	22 (43.14)	29 (34.12)	25 (29.41)	23 (32.39)	25 (45.45)	21 (55.26)	10 (50)
Aberrant motor behaviour	27 (24.32)	22 (19.82)	34 (34.69)	39 (42.86)	32 (41.56)	18 (35.29)	28 (32.94)	28 (32.94)	23 (32.39)	22 (40)	14 (36.84)	6 (30)
Nighttime behaviour disturbances	30 (27.03)	28 (25.23)	27 (27.55)	23 (25.56)	21 (28.38)	12 (23.53)	46 (54.76)	34 (40)	25 (35.21)	18 (32.73)	17 (44.74)	10 (50)
Appetite and eating abnormalities	38 (34.55)	40 (36.04)	24 (24.49)	29 (31.87)	22 (28.57)	11 (21.57)	29 (34.52)	21 (24.71)	18 (25.35)	18 (32.73)	7 (18.42)	6 (30)

### Functional assessment

Functional decline was assessed with the first 13 items from the Rapid Disability Rating Scale (RDRS-2) (eating, making simple food (e.g., sandwiches), cooking dinner and adhering to a diet, mobilization, daily personal care, personal hygiene, bathing, dressing, toilet usage, telephone usage, buying food and other necessary items, handling money, having a financial overview plan ahead, and taking medications) (19–21). Severity of functional decline on each item was scored from 1 to 4 (can perform the task alone = 1, with some help = 2, with a lot of help = 3, and cannot perform = 4), with a higher score indicating worse function. ADL scores were obtained using the sum of the products between each ADL item by its severity score (1–4) and dividing this by the number of total items of the scale (i.e., [Eating Item × Eating Severity +…]/ 13).

### Assessment of neuropsychiatric symptoms

We used 12 items from the validated Norwegian version of the 12-question Neuropsychiatric Inventory (NPI) to interview family members or caregivers (22, 23). NPI scores were evaluated annually across the 5-year follow-up time. The total score of each NPI item (i.e., hallucinations, delusions, agitation/aggression, dysphoria/depression, anxiety, irritability, disinhibition, euphoria, apathy, aberrant motor behavior, sleep, and nighttime behavior change, appetite, and eating change) was obtained by multiplying the frequency (ranging from rarely = 1 to very often = 4) by the severity (ranging from mild = 1 to severe =3) of each NPS, with a maximum score of 12. The absence of symptoms in a certain NPI item was coded as 0. The total NPI was also computed by summing the total scores of the 12 items, with a maximum NPI total score of 144.

### Confounding variables

We included demographic factors, such as sex and age. The number of comorbidities was assessed using the Cumulative Illness Rating Scale (CIRS) (26) based on patient and informant reports. Cognition was evaluated using the MMSE (12).

### Statistical analysis

#### Baseline differences between DLB and AD

A descriptive analysis was conducted for all variables included, using means with standard deviations (SD) for continuous variables, and counts and percentages for discrete ones. This analysis was stratified for each study time point, from baseline to wave 5; including number of individuals for each assessment to describe attrition. To provide a picture on how function changed with time, each symptom was plotted according to its presence (i.e., yes or no) and respective RDRS-2 scores at all waves. Plots were fitted with fractional polynomial to visualize the sample estimation. A mixed effect model (random slope) was used to test the association of the trajectory of the complete NPI scores across waves with the RDSR-2 score for all the waves. Age and sex were entered as fixed terms, while the rest (see below) were random. Beta coefficients with 95% confidence intervals (CI) are presented as a measure of the strength of association; models were adjusted for age, sex, CIRS, MMSE, and a time/squared time * NPI score was included; if the term was significant, estimates of models adjusted for interaction were included, if interaction term was not significant, beta coefficients from the model without the interaction are presented (see below). Both NPI and RSDR-2 scores were standardized (i.e., *z*-scores) for comparison purposes into a fitted plot showing the relation of their trajectories. Finally, in a similar fashion to that of the complete NPI scores, individual dichotomized symptoms from the NPI, mixed effects model were used, and adjusted for co-variates, including the interaction terms with time/squared time, using adjustment for multiple comparisons. Al the analyses were performed with STATA 17.

## Results

Table 1 presents the sample characteristics of the AD and LBD groups, including the demographic variables and distributions across study time points. Our results indicate a higher male prevalence in the LBD group at baseline, with similar age at baseline (AD 75.2 ± 7.72 vs. LBD 75.42 ± 6.9) and global cognitive function by the MMSE (AD 23.6 ± 2.31 vs. LBD 23.65 ± 3.2).

**Figure 1:**
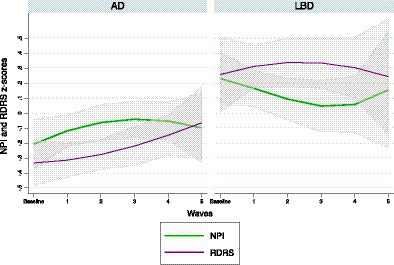
NPI and RDRS *z*-scores with 95% confidence intervals by wave and stratified by diagnosis.

Table 2 presents the analysis of the influence of the NPI total score on the functional decline trajectories. Overall, total NPI was significantly associated with RDRS-2 trajectories in both the AD (beta = 0.046; *p* < 0.001) and LBD group (beta = 0.07; *p* <  0.001). The higher the NPI total score (higher NPS burden), the higher the RDRS-2 score (worse ADL performance). Figure 1 shows the trajectories of total NPI and RDRS-2 scores throughout the follow-up, with a more pronounced functional decline and increased total burden of NPS in the LBD group. Regarding the Interaction with time, in LBD, the total NPI score significantly decreases over the course of follow-up.

**Table 2:** Linear mixed models for functional trajectories explained by total NPI score and adjusting covariates, results by diagnosis.

	AD			LBD		
	Beta coefficients	Confidence intervals	Value of *p*	Beta coefficients	Confidence intervals	Value of *p*
NPI Total	0.046	(0.026–0.067)	0.000	0.07	(0.032–0.107)	<0.001
Age	0.009	(0.00–0.016)	0.011	0.013	(−0.001–0.028)	0.07
Sex	−0.152	(−0.266 – −0.037)	0.009	−0.113	(−0.314–0.087)	0.268
CIRS	0.027	(0.003–0.050)	0.273	−0.011	(−0.05–0.028)	0.57
MMSE	−0.067	(−0.074– −0.059)	0.000	−0.051	(−0.063 – −0.04)	<0.001
NPI Total *time	−0.001	(−0.0009–0.008)	0.939	−0.017	(−0.031 – −0.004)	0.011
NPI Total *squared time	0.0001	(−0.017–0.017)	0.987	0.0005	(−0.018–0.019)	0.956

The symbol “*” represents the multiplication sign for the interaction between “NPI Total” and “squared time” and is not a note.

Table 3 shows complete results for all NPI items, the size of the effect of the associations, and their value of ps. Overall, in individuals with AD, the presence of hallucinations, aggression, depression, anxiety, apathy, disinhibition, aberrant motor behavior, nighttime behavior disturbances, and abnormal eating patterns were significantly associated with an increased decline in ADL over a 5-year follow-up when compared with those without the symptom. Similarly, the presence of irritability and aberrant motor behavior in those with LBD exhibited significant associations with functional decline trajectories. The analysis showed that the influence of time on depression/dysphoria (Beta = 0.562, *p* = 0.017) was significant in the AD group. Additionally, irritability (Beta = 0.276, *p* = 0.006) and motor behavior (Beta = 0.23, *p* < 0.001) also had significant associations for LBD.

**Table 3:** Linear mixed models for functional trajectories explained by each NPI symptom and the adjusting variables, results by diagnosis.

	AD			LBD		
	Beta coefficients	Confidence intervals	Value of *p*	Beta coefficients	Confidence intervals	Value of *p*
Delusions	0.014	(−0.09–0.12)	0.802	0.095	(−0.049–0.241)	0.112
Hallucinations	0.211	(0.07–0.353)	0.003	0.092	(−0.048–0.233)	0.200
Agitation/agression*	0.204	(0.095–0.055)	0.001	−0.423	(−0.937–0.089)	0.106
Depression/dysphoria**	0.562	(0.098–1.025)	0.017	0.129	(−0.010–0.269)	0.070
Anxiety	0.103	(−0.011–0.207)	0.053	0.077	(−0.064–0.220)	0.284
Euphoria	0.093	(−0.092–0.278)	0.326	0.209	(−0.108–0.526)	0.197
Apathy	0.152	(0.052–0.252)	0.003	0.068	(−0.071–0.209)	0.335
Disinhibition	0.118	(0.025–0.234)	0.045	0.083	(−0.083–0.250)	0.325
Irritability*	0.029	(−0.071–0.130)	0.572	0.276	(0.078–0.474)	0.006
Aberrant motor behavior*	0.147	(0.036–0.259)	0.009	0.23	(0.189–0.289)	<0.001
Nighttime behavior disturbances	0.116	(0.011–0.232)	0.048	0.028	(−0.113–0.171)	0.693
Appetite and eating abnormalities	0.166	(0.057–0.275)	0.003	0.106	(−0.051–0.264)	0.188

^*^Time x NPI item interaction (*p* < 0.05) in LBD.

^**^Squared time x NPI item interaction (*p* < 0.05) in AD.

**Figure 2:**
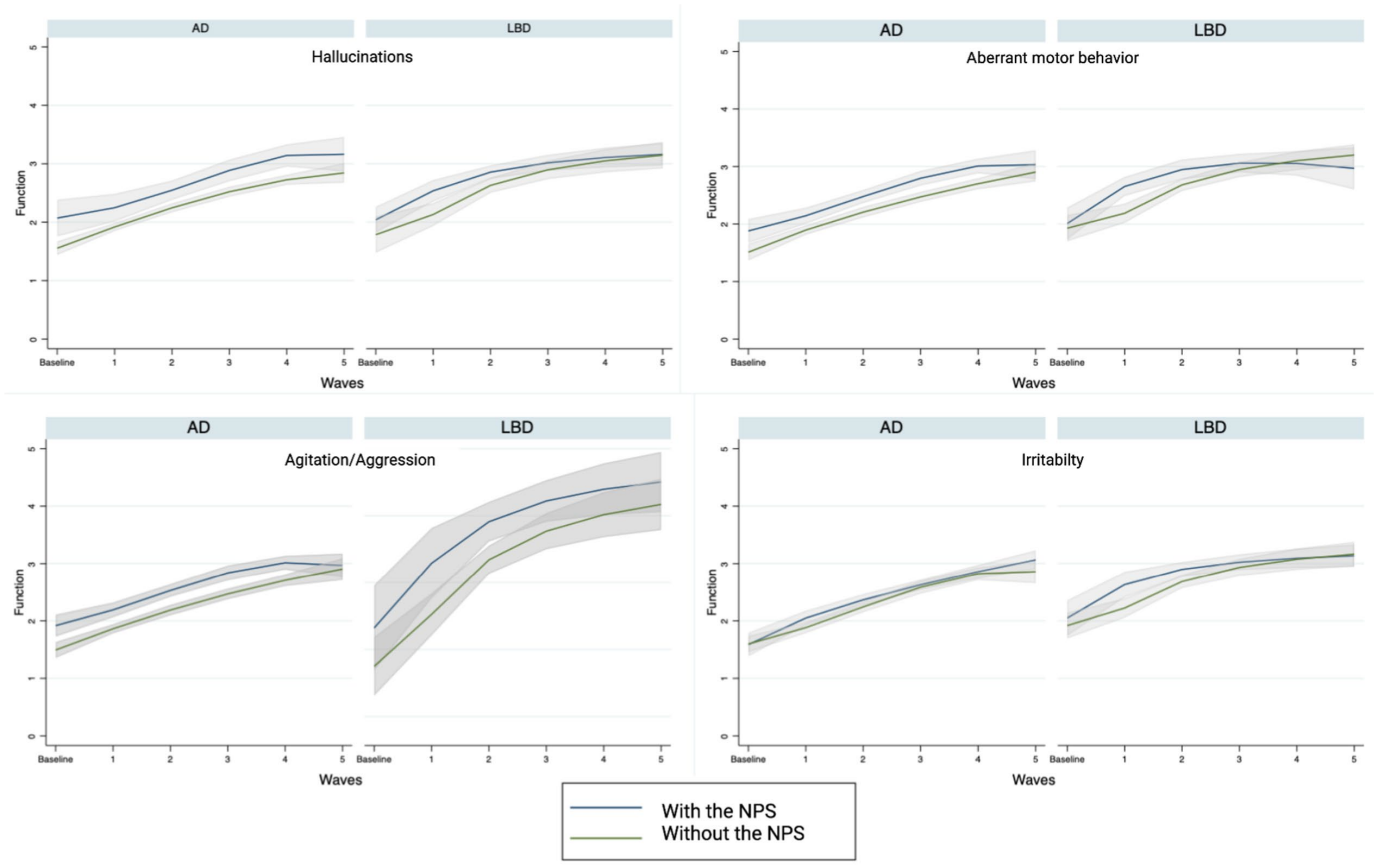
Functional trajectories with and without specific NPS.

In Figure 2, we present the trajectories of functional decline in the NPS during the follow-up time points, stratified into groups by the presence or absence of the studied symptom.

## Discussion

Specific NPS significantly affect functional decline trajectories in patients with AD and LBD. Moreover, the influence of some NPS on disease progression, in terms of functional decline, varied between these two neurodegenerative conditions. Thus, in AD, hallucinations, aggression, depression, anxiety, apathy, disinhibition, aberrant motor behavior, nighttime behavior disturbances, and abnormal eating patterns were found to be associated with the 5-year decline in the performance of ADL, and irritability and aberrant motor behavior in those with LBD.

NPS have also previously been associated with functional impairment (7, 24). however, most of the research available has been performed in the AD population, and scarce evidence is available regarding LBD patients. NPS –in particular psychotic symptoms and aggressive behaviors– are strongly associated with adverse outcomes, such as higher rates of disability, caregivers’ burnout, and nursing home placement (25, 26). For example, reports show an increased early cognitive and functional deterioration over time in people with psychotic symptoms in AD (27, 28). Psychotic symptoms can significantly disrupt an individual’s daily functioning, particularly when they impact safety and their ability to interact with their environment, family, and social relationships. These symptoms are often very noticeable and can lead to an increased use of multiple medications, the development of comorbidities, limited mobility, changes in dietary habits, the need for a caregiver, and even requiring nursing home placement; a feasible explanation for our findings (4–6, 24, 29, 30).

NPS are prevalent in older adults and can substantially affect their functional trajectories. You et al. (31) reported that apathy and nighttime behaviors predict functional trajectories in AD (31). Accordingly, our study also found that sleep and nighttime behavioral symptoms significantly influence functional decline in patients with AD. Sleep disturbances are markers for the diagnosis of several neurodegenerative disorders, such as LBD. However, in AD, sleep and nighttime behavioral symptoms could serve as potential markers for a more pronounced functional decline. In addition to functional decline, other relevant outcomes have been associated with sleep and nighttime behavioral symptoms. This was found in a publication by Hope et al., who reported that nighttime behaviors, including sleep disturbances, were predictors of institutionalization in patients with DLB within 1 year (32).

The severity of depression has also been related to functional disability in previous research on the dementia population (33). In our study, depression was frequent and significant for AD’s functional decline. In addition to the spectrum of psychotic symptoms, other NPS may also affect functional decline trajectories. Depression and dysphoria may lead to social isolation, immobility, sarcopenia, falls, frailty, and greater disability. This is thought to be due to the fact that NPS are associated with greater neuronal damage and more widespread neuropathology in AD (34).

In contrast, in the case of LBD, the impact of NPS on the functional prognosis may be less pronounced. Although NPS are still common in LBD and can contribute to functional decline, they may not have the same degree of impact on disease progression as in AD. The latter might be explained since LBD is characterized by more variable clinical features, including fluctuations in cognition and motor symptoms, which may lead to fluctuating trajectories of decline and NPS (3). Despite our findings on the impact of NPS on functional trajectories, this relationship between is likely to be complex and multifactorial, also influenced by a range of biological and environmental factors (35).

It is important to note that we are aware of the potential overlapping between symptoms among different NPI items (e.g., depression with anxiety and apathy) (36). When patients present with dementia, this makes it difficult to isolate the effects of individual symptoms on the disease progression. Future research should continue to investigate the relationship between different symptoms and their impact on patients’ overall well-being, while considering the potential interaction between symptoms.

We are aware that our research may have a potential recruitment bias because of the referrals of primary care patients, which may have led to an increased number of patients with complicated dementia or NPS. However, GPs were encouraged to refer patients with suspected dementia, and patients were recruited from psychiatric, neurologic, and geriatric clinics. In addition, we used the NPI to assess NPS, which does not capture the full spectrum of NPS and is entirely based on caregiver reports, and thus, not the subjective experiences of the patients. In addition, it was not possible to consider the non-pharmacological or pharmacological concomitant therapies that were administered to the patients. We are aware that this may have influenced the functional outcomes. Euphoria associations were not significant; however, this was an infrequent symptom, and this finding must be interpreted with caution. Finally, as expected, mortality rates were –as expected– very high, particularly in LBD, which may have confounded the observed course of functional performance. In contrast, the internal validity of our conclusions is supported by the strengths of the design, including a long follow-up time, yearly assessments with structured instruments, and high completeness of data. The diagnostic procedures were rigorous, and high accuracy was demonstrated with neuropathological diagnosis (37). As this is one of the very few long-term studies assessing daily functioning in LBD, we encourage further research to examine the external validity and reproducibility of our conclusions in the LBD population.

Our group has previously reported that NPS fluctuates over time across the progression of AD and LBD (38), which could alter the total prediction of symptoms trajectory. However, it is worth noting that this research sheds new light on potential targets for interventions that when detected in mild stages could impact and help improve an individual’s functional prognosis. This study highlights the importance of assessing the presence of NPS in patients with LBD and AD as they can significantly impact the functional trajectory of these patients. Identifying the target NPS could make clinicians aware of specific symptoms potentially associated with more pronounced functional decline, thus prioritizing their management. The present findings have important implications as NPS are common, underdiagnosed, or in many cases, assumed as normal (39). Little research on this matter is currently available. Our results thus would appear to be of clinical and scientific interest, particularly in LBD research.

NPI are extremely difficult symptoms to manage. Antipsychotic medications, sedative-hypnotic, antidepressants and other psychotropics drugs are routinely used to control NPI in most cases in clinical practice, even off-label. These medications are likely to cause parkinsonism, sedation, motor retardation thus leading to reduced mobility, increased risk of falls, and disability. Because of the effects of these medications, NPI may also get worse. All these events are definitely likely to influence trajectories of functional decline among patients with dementia. It is difficult to disentangle the role of the symptoms on influencing the risk of disability over time without taking into account the role of medications. Also, physical restraints are often adopted to manage NPI, especially for extremely severe symptoms and they should be also considered as potential contributor to trajectories of functional decline in these patients. Failure to control for these factors may eventually influence the validity of findings and conclusions.

## Data availability statement

The raw data supporting the conclusions of this article will be made available by the authors, without undue reservation.

## Ethics statement

The studies involving humans were approved by the Regional Ethics Committe of Stavanger. The studies were conducted in accordance with the local legislation and institutional requirements. Written informed consent for participation in this study was provided by the participants’ legal guardians/next of kin.

## Author contributions

MUPZ: Validation, Writing – review & editing. MB: Conceptualization, Data curation, Investigation, Methodology, Project administration, Writing – original draft. KB: Conceptualization, Supervision, Validation, Writing – review & editing. EG-C: Data curation, Visualization, Writing – review & editing. AJ-J: Data curation, Formal analysis, Validation, Writing – review & editing. CR-O: Supervision, Writing – review & editing. JPB: Data curation, Formal analysis, Methodology, Writing – review & editing. HS: Supervision, Validation, Writing – review & editing. AV-M: Supervision, Writing – review & editing. DA: Conceptualization, Funding acquisition, Resources, Supervision, Writing – review & editing.
